# Airway Management in Complex Maxillofacial Trauma: Evaluating the Role of Submental Intubation as a Viable Alternative to Tracheostomy

**DOI:** 10.3390/cmtr18010021

**Published:** 2025-03-17

**Authors:** Giulio Cirignaco, Gabriele Monarchi, Lisa Catarzi, Mariagrazia Paglianiti, Enrico Betti, Umberto Committeri, Alberto Bianchi, Paolo Balercia, Giuseppe Consorti

**Affiliations:** 1 Department of Medicine, Section of Maxillo-Facial Surgery, University of Siena, Viale Bracci, 53100 Siena, Italy; g.cirignaco@student.unisi.it (G.C.); gabriele.monarchi@gmail.com (G.M.); lisa.catarzi@gmail.com (L.C.); mgpaglianiti@gmail.com (M.P.); 2 Division of Maxillofacial Surgery, Department of Neurological Sciences, Marche University Hospitals-Umberto I, 60126 Ancona, Italy; enrico.betti@ospedaliriuniti.marche.it (E.B.); paolo.balercia@ospedaliriuniti.marche.it (P.B.); 3 Division of Maxillofacial Surgery, “Santa Maria” Hospital, V.le Tristano di Joannuccio, 05100 Terni, Italy; umbertocommitteri@gmail.com; 4 Department of General Surgery and Medical-Surgical Specialties, University of Catania, 95123 Catania, Italy; alberto.bianchi@unict.it

**Keywords:** maxillofacial fracture, submental intubation, tracheostomy, airway management, complications, hospitalization days

## Abstract

Airway management in maxillofacial trauma is a critical and complex challenge, requiring both secure ventilation and optimal surgical access while minimizing risks to vital structures. This study evaluated the efficacy of submental intubation (SMI) as a minimally invasive alternative to tracheostomy in patients with complex maxillofacial fractures. A retrospective analysis of 52 patients treated between 2015 and 2023 was conducted by comparing clinical outcomes between those who underwent SMI (n = 26) and those who underwent tracheostomy (n = 26). The duration of hospitalization, infection rates, and perioperative complications were assessed using t-tests, chi-square tests, and multivariate regression. Results indicated that SMI was associated with significantly shorter hospital stays (11.15 ± 3.29 vs. 23.96 ± 6.47 days, *p* < 0.001) and lower infection rates (3.8% vs. 30.8%, *p* = 0.028). Additionally, the SMI group demonstrated fewer intraoperative (*p* = 0.049) and postoperative complications (*p* = 0.037). Multivariate analysis identified tracheostomy as an independent predictor of prolonged hospitalization and increased complications. These findings support SMI as a safe and effective alternative to tracheostomy for short-term airway management in maxillofacial trauma, providing a shorter recovery period and fewer complications. Therefore, prospective studies with larger cohorts are warranted to confirm these results and establish comprehensive guidelines.

## 1. Introduction

Airway management is a crucial aspect of modern surgical and anesthetic care, especially in the treatment of complex maxillofacial fractures [1].

These injuries pose challenges due to their proximity to vital structures and the necessity for precise surgical intervention to restore function and esthetics. Achieving an adequate surgical field and maxillomandibular fixation can further complicate airway management.

Key surgical concerns in facial fracture management include maintaining dental occlusion when possible, supporting facial tissues, and preserving nasal projection and airway patency [2]. Effective airway management is essential to achieving these objectives and ensuring successful outcomes. The anatomical complexity of maxillofacial trauma requires careful planning to ensure both ventilation and surgical accessibility. Inadequate airway protection and poor ventilation contribute to 16% of trauma-related deaths [1].

Traditional approaches to airway management in maxillofacial trauma usually include naso- or orotracheal intubation. Nasotracheal intubation, along with intermaxillary fixation, is the first option in isolated mandibular fracture or with concomitant midfacial fracture reduction [3]; while effective in many cases, it is contraindicated in scenarios such as complex midface fractures or naso-orbital-ethmoid (NOE) and anterior skull base fractures [1]. In the case of anterior skull base fracture, nasotracheal intubation can create a false passage, risking intracranial tube penetration and potential Cerebrospinal Fluid (CSF) leakage or meningitis [4].

Tracheostomy is typically used in patients with severe systemic injuries requiring prolonged mechanical ventilation, extensive comorbidities, or difficulties in weaning from ventilatory support. It provides a secure airway and allows for unrestricted surgical access [5]. However, it has notable drawbacks and a higher risk of complications, such as surgical emphysema, pneumothorax, infection, cervical vessel injury, and scarring [1,3,6]. Additionally, tracheostomy often necessitates prolonged postoperative care, which may increase healthcare costs, patient discomfort, and risk of infection [5,7].

Initially described by Hernández Altemir, submental intubation involves a midline incision and offers a less invasive alternative to tracheostomy for short-term airway management [8]. After standard orotracheal intubation is performed, the patient’s head and neck are positioned to optimize access to the submental region. A small 1.5–2 cm transverse incision is made immediately posterior to the mandibular symphysis (or slightly paramedian when indicated). Care is taken to identify and protect the sublingual glands, Wharton’s duct, and lingual nerves. Dissection is then performed smoothly, closely following the lingual cortical plate using a Klemmer until the oral cavity is reached. Once the passage has been created, the pilot balloon is inserted first, followed by the armored endotracheal tube. Once the tube is externalized, it is reconnected to the breathing circuit. The final position is checked via auscultation and capnography to ensure adequate ventilation [9,10,11,12]. Throughout the procedure, sterile precautions are maintained, and the incision is irrigated with an antiseptic solution. After completion of the maxillofacial procedure and stabilization of fractures (including any required maxillomandibular fixation), the tube is removed first, followed by the pilot balloon, thus reversing the order of insertion. In our practice, only a cutaneous closure is performed, with no intraoral suturing required [5]. The incision site is selected based on patient-specific anatomical factors, such as scar tissue, sublingual gland prominence, and mandibular morphology. The potential implications of these modifications include reduced risk of postoperative edema and better cosmetic outcomes, although with a potentially increased risk of hematoma formation. This approach allows intermaxillary fixation (IMF) and the treatment of NOE and midfacial fractures with the same advantages as naso- or orotracheal intubation.

Submental intubation has become increasingly recognized for its lower complication rates, shorter operative times, and improved patient satisfaction compared to tracheostomy [13,14]. However, the technique has limitations, such as the risk of scarring at the incision site and the need for operator expertise to prevent complications like tube damage, sublingual/submandibular gland injury, and lingual nerve injury [4,15,16].

Despite its advantages, submental intubation remains underutilized as many clinicians prefer tracheostomy due to its established protocols and suitability for prolonged ventilation [14]. This dichotomy highlights the need for a comprehensive evaluation of these techniques to guide clinical decision-making, particularly in complex maxillofacial trauma cases where airway management is critical. This study compares the safety, efficacy, and clinical outcomes of submental intubation versus tracheostomy in maxillofacial trauma, providing evidence-based recommendations that highlight the strengths and limitations of each technique.

Although previous studies have assessed submental intubation and tracheostomy [1,15,17,18,19,20], few have focused on hospital length of stay and infection rates as primary outcomes [5]. Our analysis highlights the importance of tailoring airway management strategies to patient-specific factors to refine decision-making in complex maxillofacial trauma. This approach enables a more nuanced comparison between submental intubation and tracheostomy in complex maxillofacial trauma. Our retrospective analysis adds to the literature by emphasizing these parameters and by examining how patient-level factors (such as injury severity and comorbidities) may modify the relative benefits of each technique. Moreover, based on both the findings of the present study and our institutional experience, we aim to propose practical guidelines for airway management in maxillofacial trauma to be validated in the future.

## 2. Materials and Methods

### 2.1. Study Design

This retrospective study included a cohort of patients who underwent surgical treatment for maxillofacial fractures at the Maxillofacial Surgery Unit of Azienda Ospedaliera Ospedali Riuniti in Ancona (Marche, Italy) between January 2015 and December 2023. Patients were selected from the hospital’s database, allowing for a comprehensive analysis of clinical outcomes in this population.

The study population dataset was divided into two groups based on the surgical approach for maxillofacial trauma management: the SMI technique group and the tracheostomy group.

Patient data, including age, gender, fracture type, and reduction method, were collected from the hospital’s medical records and operating room activity recording software (Ormaweb^®^, Version 6.2.1 Dedalus Italia Spa, Firenze, Italy). All data were recorded and tabulated using Microsoft Excel (Version 16.63.1; Microsoft Corporation, Redmond, WA, USA). No personal identifiers, such as names or surnames, were collected at any stage of this study. All surgical procedures were performed exclusively by experienced maxillofacial surgeons, ensuring consistency in the treatment approach and minimizing variability in outcomes associated with trainees or less experienced operators [21].

Inclusion criteria covered patients with radiologically confirmed fractures involving the mandible, maxilla, NOE complex, or anterior skull base who required surgical airway management. All included patients underwent either tracheostomy or submental intubation and required occlusal control during surgical procedures. The exclusion criteria included the following: patients with incomplete data records; patients with anamnesis of previous facial fractures surgically treated; patients urgently tracheostomized for reasons other than treating fracture (e.g., respiratory distress); patients who did not provide consent to inclusion in the study; patients with incomplete data charts.

The choice between submental intubation and tracheostomy was based on clinical factors, including the need for prolonged ventilation and the severity of associated injuries. No randomization was applied, and institutional protocols, surgeon experience, and patient-specific factors influenced the decision-making process.

### 2.2. Variables

Standardized data were gathered for each patient in the database: gender, age, cause, type of fracture, cause of fracture, associated injuries, comorbidities, type of airway management, complication related to the airway management procedure itself, and length of hospital stay measured from the date of hospital admission to the date of discharge.

The anatomic fracture location was assessed using Computer Tomography (CT) scans performed during the diagnostic evaluation in the emergency department of our hospital or an external facility. The fractures were classified according to the AO-Craniomaxillofacial trauma criteria [22] into the following categories: frontal sinus, nose, NOE complex, zygomaticomaxillary complex (COMZ), maxilla, mandible, anterior skull base. Fracture associations were also assessed, particularly in cases of panfacial trauma where multiple sites were involved. Work-related injuries included facial trauma sustained in occupational settings, primarily due to high-energy mechanisms such as falls from height, impact from heavy machinery, and construction-related accidents. These injuries were distinguished from low-energy occupational incidents, such as minor falls or accidental blunt trauma, which were categorized separately.

### 2.3. Statistical Analysis

Statistical analysis was performed to evaluate differences in clinical outcomes, focusing on hospitalization duration, infection rates, intraoperative and postoperative complications, and their respective predictors. Descriptive statistics were employed to provide a comprehensive summary of demographic and clinical characteristics. Means, medians, standard deviations, and ranges were calculated for continuous variables, while categorical variables were expressed as frequencies and percentages. Inferential analyses included independent *t*-tests for comparing continuous variables such as hospitalization duration, while chi-square tests were applied to evaluate associations between categorical variables, including complication rates and procedure types. Assumptions of normality and homogeneity of variances were tested using the Shapiro–Wilk and Levene’s tests, respectively. Logistic regression was employed to identify predictors of complications and infections, while linear regression was used to assess factors influencing hospitalization duration. Additionally, multivariate analyses were performed, as follows: logistic regression to identify independent predictors of complications and infections; linear regression to explore factors influencing hospitalization duration. Statistical significance was set at *p* < 0.05, with 95% confidence intervals provided where appropriate. Statistical analyses were performed using Jamovi Statistics version 29.0.1.0 (IBM, ARMONK, New York, NY, USA).

## 3. Results

### 3.1. Demographic and Clinical Characteristics

Of the 105 patients assessed, 52 met the inclusion criteria and were included in the study. The study population was divided into two groups based on the surgical approach for maxillofacial trauma management: the SMI technique group (n = 26) and the tracheostomy group (n = 26).

The total patient cohort included 40 males (76.9%) and 12 females (23.1%), with a mean age of 42.2 years (SD = 17.1). However, within the subgroups, gender distribution differed: 84.6% of the SMI group were male (n = 22), compared to 69.2% in the tracheostomy group (n = 18), but this difference was not statistically significant (*p* = 0.323). Conversely, the mean age was significantly higher in the tracheostomy group (45 ± 17.4 years) than in the SMI group (39 ± 16.3 years, *p* < 0.001).

Regarding associated injuries, the tracheostomy group had a higher prevalence of concomitant systemic trauma compared to the SMI group. Specifically, patients undergoing tracheostomy exhibited injuries such as frontal brain hematoma (19.2%, n = 5), post-traumatic epilepsy (11.5%, n = 3), and thoracic fractures (including rib and vertebral fractures) in 15.4% of cases (n = 4). Additionally, upper limb fractures were observed in 7.7% of patients (n = 2), and complex injuries involving multiple systems (e.g., pulmonary embolism and cervical fractures) were present in 11.5% (n = 3). In contrast, the SMI group demonstrated fewer associated injuries overall, with 7.7% (n = 2) exhibiting minor rib fractures and 11.5% (n = 3) showing isolated brain contusions.

In terms of fracture types, patients in the tracheostomy group most frequently presented with bilateral COMZ fractures (38.5%, n = 10), Le Fort I and II fractures (34.6%, n = 9), and anterior skull base fractures (15.4%, n = 4). Conversely, the SMI group exhibited a higher prevalence of NOE complex fractures (30.8%, n = 8), nasal bone fractures (23.1%, n = 6), and Le Fort III fractures (11.5%, n = 3). Both groups demonstrated a similar incidence of mandibular fractures, with parasymphysis and condylar fractures observed in 11.5% (n = 3) of cases in each group.

The most common fracture combinations were Le Fort I/II fractures associated with mandibular fractures (17.3%, n = 9), bilateral COMZ fractures with NOE involvement (21.1%, n = 11), and anterior skull base fractures combined with NOE involvement (11.5%, n = 6).

As for the causes of trauma, road collisions accounted for the majority of cases (53.8%, n = 28), followed by aggressions (23.1%, n = 12), work-related injuries (11.5%, n = 6), and other causes such as falls from heights over two meters (11.5%, n = 6). These etiologies were similarly distributed between the two groups, with no significant differences in trauma mechanisms observed (*p*-values ranging from 0.639 to 0.755). A summary of demographic and clinical characteristics of both groups is provided in Table 1.

### 3.2. Hospitalization Duration

Hospitalization duration was assessed in two ways: as the total length of stay from admission to discharge, and as the time from admission to surgery, thereby providing additional insight into preoperative management. Patients in the tracheostomy group had an average total hospital stay of 23.96 days (SD = 6.47), which included a mean preoperative period of 5.2 days (SD = 2.1) before surgery. In contrast, the SMI group experienced a significantly shorter overall stay, with a mean of 11.15 days (SD = 3.29) and a mean preoperative period of 2.8 days (SD = 1.4) (t = −8.99, *p* < 0.001, 95% CI: −15.6 to −9.7). A longer preoperative stay observed in the tracheostomy group was associated with the complexity of injuries that required multidisciplinary stabilization prior to surgery.

It is noteworthy that the differences in preoperative waiting times were not solely related to clinical factors or injury severity. In our institution, such delays are also influenced by logistical and organizational issues, including operating room availability and the coordination of multidisciplinary teams. These institutional factors can extend the time from admission to surgery, particularly in cases requiring stabilization of other injuries

**Figure 1:**
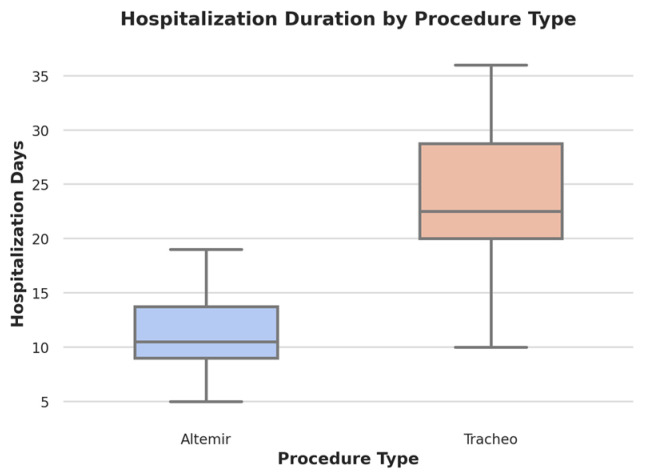
Boxplot illustrating hospitalization duration by procedure type. The Altermir technique (blue) shows a shorter hospitalization duration with a mean of 11.15 days (SD = 3.29), while tracheostomy (pink) patients experienced significantly longer hospital stays, with a mean of 23.96 days (SD = 6.47) (*p* < 0.001). The plot highlights the distribution, median, and variability of hospitalization days for each surgical approach.

Hospitalization was significantly longer in the tracheostomy group, particularly in patients with systemic injuries such as pulmonary embolism (mean: 32 days, 95% CI: 27.8–36.2) and multiple fractures (mean: 28 days, 95% CI: 24.1–31.9). This difference was statistically significant (t = −8.99, *p* < 0.001). However, while airway management appears to be associated with different hospitalization durations, this study does not establish a direct causal relationship. Other factors, including the severity of associated injuries, perioperative care, and rehabilitation protocols, may have also contributed to the observed differences (Figure 1).

### 3.3. Infection Rates

Postoperative infections occurred significantly more frequently in the tracheostomy group, with 30.8% of patients (n = 8) experiencing infections compared to only 3.8% of patients (n = 1) in the SMI group (χ^2^ = 4.84, *p* = 0.028; OR = 10.4, 95% CI: 1.1 to 91.3). Tracheal contamination was the most common cause of infection in the tracheostomy group, accounting for 50% of cases (n = 4). In this group, infections included tracheal colonization (n = 4), pneumonia (n = 3), and wound site infections (n = 1), whereas the single case in the SMI group was a localized wound infection that resolved with conservative treatment.

### 3.4. Intraoperative and Postoperative

Intraoperative complications were observed in 19.2% (n = 5) of the tracheostomy group and 7.7% (n = 2) of the SMI group. Specific complications in the tracheostomy group included subcutaneous emphysema (7.7%, n = 2), thyroid gland injury (3.8%, n = 1), and hemorrhage (7.7%, n = 2), while the SMI group experienced balloon damage (3.8%, n = 1) and hematoma formation (3.8%, n = 1). The association between procedure type and intraoperative complications was statistically significant (χ^2^ = 3.89, *p* = 0.049 OR = 2.96, 95% CI: 0.94 to 9.31), emphasizing the increased intraoperative risks associated with tracheostomy.

Postoperative complications were similarly higher in the tracheostomy group, with 23.1% of patients (n = 6) affected compared to 11.5% (n = 3) in the SMI group. Tracheostomy-related complications included tracheomalacia (11.5%, n = 3), scar dehiscence (7.7%, n = 2), and submental swelling (3.8%, n = 1), whereas the SMI group included submental swelling (7.7%, n = 2) and hypertrophic scars (3.8%, n = 1). The difference in postoperative complication rates between the two groups was statistically significant (χ^2^ = 4.36, *p* = 0.037 OR = 2.35, 95% CI: 0.72 to 7.61), further underscoring the increased postoperative risks in patients undergoing tracheostomy. A direct correlation was observed between the presence of intraoperative complications and prolonged hospital stays, with patients experiencing such events requiring an additional mean duration of 6.5 days (95% CI: 4.2 to 8.9, *p* = 0.041).

### 3.5. Predictors of Prolonged Hospitalization and Complications

To identify predictors of clinical outcomes, multivariate analyses were also performed: linear regression analysis revealed that infections were the strongest predictor of prolonged hospitalization, with an estimated increase of 15.62 days (95% CI: 10.4 to 20.8 *p* = 0.022) in patients with infections. Additionally, the procedure type was defined as an independent contributor to hospitalization duration, with tracheostomy adding an average of 8.93 days to the hospital stay (95% CI: 5.6 to 12.2, *p* = 0.041). Regarding infections and complications, logistic regression analysis demonstrated that tracheostomy was a significant predictor of postoperative infections and complications. Logistic regression analysis demonstrated that tracheostomy was significantly associated with a higher likelihood of both intraoperative complications (OR = 2.96, *p* = 0.031; 95% CI: 1.02 to 8.59) and postoperative infections (OR = 3.21, *p* = 0.027; 95% CI: 1.17 to 8.80). Similarly, patients with complex fractures, such as NOE and anterior skull base injuries, were more likely to experience prolonged hospital stays (mean: 28 days, 95% CI: 24.2 to 31.8, *p* = 0.034) and higher rates of infection (21.7%, 95% CI: 12.8 to 31.6). In contrast, patients with isolated mandibular fractures had shorter hospital stays (mean: 8.5 days, 95% CI: 7.2 to 9.8, *p* = 0.027). The comprehensive results are summarized in Table 2.

## 4. Discussion

This study aimed to evaluate and compare the outcomes of submental intubation and tracheostomy in the management of maxillofacial trauma. By specifically analyzing hospitalization duration, infection rates, and complications, our goal was to show both the advantages and disadvantages of each technique and, eventually, guide the choice of airway management in complex surgical scenarios.

A key finding of this study is that patients who underwent SMI had a significantly shorter average hospital stay (11.15 days) than those managed with tracheostomy (23.96 days, *p* < 0.001). These findings are consistent with prior research such as the study by Mahajan et al. [13] who highlighted its association with shorter recovery times. Similarly, Mishra et al. [11] reported comparable benefits of SMI. The prolonged length of stay observed in tracheostomy patients can largely be attributed to increased postoperative care requirements, although tracheostomy remains essential for cases requiring prolonged ventilation [3,6,23].

The higher infection rate in the tracheostomy group (30.8%) compared to the SMI group (3.8%, *p* = 0.028) further underscores the risks associated with tracheostomy. Tracheostomy-related infections were primarily attributed to tracheal contamination and prolonged exposure, consistent with the observations by Jundt et al. [4]. The isolated infection in the submental intubation group was attributed to localized wound dehiscence, a complication that is typically resolved with conservative management. However, infection rates vary across institutions, suggesting that operator experience and perioperative protocols significantly impact outcomes [6].

Complication rates, both intraoperative and postoperative, further emphasized the advantages of submental intubation. Intraoperative complications occurred in 19.2% of tracheostomy cases compared to 7.7% in submental intubation cases (*p* = 0.049). Similarly, postoperative complications were observed in 23.1% of the tracheostomy group compared to 11.5% in the submental intubation group (*p* = 0.037). These complications in the tracheostomy group underscore the invasiveness and technical challenges of the procedure as previously reported by Barak et al. [24] and Halum et al. [25]. By contrast, complications in the submental intubation group, such as hypertrophic scars and localized swelling, were mild and self-limiting, as reported by Lima et al. [26] and Kumar et al. [27].

Notably, multivariate analyses revealed that infections were the strongest predictor of prolonged hospitalization, adding an estimated 15.62 days (*p* = 0.022).

While the procedure type was initially significant in bivariate analysis, its direct impact decreased when controlling for confounding variables such as infections and patient demographics.

When viewed in the context of broader literature, the study reinforces the growing consensus that submental intubation is an effective and less invasive alternative to tracheostomy for short-term airway management in maxillofacial trauma [15,17,27,28]. Nevertheless, tracheostomy remains indispensable for cases requiring prolonged ventilation or for the management of complex respiratory conditions, as emphasized by Marchese et al. [29]. Furthermore, institutional protocols and operator expertise may contribute to variations in outcomes [12]. The results of this study align closely with those of Martins et al. [30] who reported similar benefits of submental intubation in reducing hospital stays and complications.

Based on our findings, we have introduced the following guidelines for our routine practice in airway management for maxillofacial trauma:

1. Midface Fractures without Extended Ventilation Needs
  - Isolated midfacial fractures (e.g., Le Fort II/III, zygomaticomaxillary complex (ZMC), NOE) in hemodynamically stable patients.
  - No severe comorbidities that would typically mandate prolonged Intensive Care Unit stay.
  - Situation where nasal intubation is contraindicated (e.g., anterior skull base fracture).

Recommended Approach: Submental intubation (SMI) is advised, as it maintains a clear surgical field, allows direct occlusal assessment, and generally avoids the complications associated with tracheostomy. It is particularly suitable when extended mechanical ventilation is unlikely.

2. Complex Panfacial Fractures or Multisystem Trauma
  - Severe craniofacial injuries involving multiple fracture sites.
  - Concomitant serious injuries (e.g., severe thoracic or spinal trauma).
  - High likelihood of prolonged sedation and mechanical ventilation.

Recommended approach: Tracheostomy remains the most secure choice in patients who need sustained mechanical ventilation or continuous sedation. Although this is a conventional strategy for severe trauma, in certain “borderline” cases—where the need for very prolonged ventilation is uncertain—a short-term trial with SMI may be considered initially to reduce tracheostomy-related complications. Should the patient’s status worsen or ventilation requirements increase, a secondary tracheostomy can then be performed.

3. Significant Pulmonary or Neurological Compromise
  - Pulmonary dysfunction that precludes early extubation (e.g., severe COPD, respiratory failure).
  - Neurological injury or status (e.g., prolonged coma) preventing safe short-term airway management.

Recommended Approach: Tracheostomy is generally indicated for these high-risk patients. However, even in cases of significant pulmonary or neurological compromise, evaluating whether only a limited period of airway control is required (e.g., a short-term sedation protocol) may open the door to an initial SMI. If long-term stability becomes necessary, conversion to tracheostomy is still an option.

Nevertheless, patient-specific factors, institutional resources, and surgical team expertise should be carefully considered in each case to optimize outcomes.

This study has limitations: First, its retrospective design prevents establishing causality and increases the risk of selection bias. Second, the relatively small sample size limits the generalizability of the findings, as does the exclusion of patients who underwent urgent tracheostomy for non-trauma-related reasons, such as severe respiratory distress or unrelated comorbidities. Third, the variability in perioperative care protocols between institutions may impact on complication rates and hospitalization durations. Fourth, several potential sources of bias must be considered in interpreting our findings such as selection bias at the time of surgery. Fifth, confounding variables such as systemic injuries, pre-existing comorbidities, and perioperative care protocols may have influenced patient outcomes. Sixth, one potential limitation is the statistically significant difference (*p* = 0.03) observed in nasal bone fracture rates between the two groups. Given the relatively small sample size and multiple comparisons in this study, there is a risk that this finding may represent a false-positive result; additionally, the apparent discrepancy in nasal bone fractures could be attributed to differences in the mechanism of injury or to unmeasured confounding variables. Seventh, variations in postoperative pain management and access to physical therapy may have influenced hospital stay duration, independent of the airway management technique.

## 5. Conclusions

In conclusion, although submental intubation was associated with shorter hospital stays and fewer complications, its use must still be carefully weighed against tracheostomy depending on patient-specific factors and the need for prolonged ventilation. The choice of airway management should consider patient-specific factors, including the need for long-term ventilation and the expertise of the surgical team. While our findings indicate an association between submental intubation and improved recovery outcomes, they do not establish causality. Because these differences may be influenced by injury severity, perioperative care, and institutional protocols, prospective randomized studies are needed to compare these airway management strategies better. Further research on patient-reported outcomes and cost-effectiveness would help optimize clinical decision-making and resource allocation.

## Figures and Tables

**Table 1:** Demographic characteristics, trauma etiology, and fracture distribution in patients treated with submental intubation compared to tracheostomy. Abbreviations: zygomaticomaxillary complex (COMZ), naso-orbital-ethmoid (NOE).

		Submental Intubation	Tracheostomy	*p*-Value
Gender	Male	22 (84.6%)	18 (69.2%)	0.323
	Female	4 (15.4%)	8 (30.8%)	0.323
Mean age ± SD		39 ± 16.3	45 ± 17.4	<0.001
	Road collision	15 (57.7%)	13 (50.0%)	0.639
Cause of trauma	Aggression	6 (23.1%)	6 (23.1%)	0.755
	Work-related injury	3 (11.5%)	3 (11.5%)	0.639
	Other causes	2 (7.7%)	4 (15.4%)	0.755
	Bilateral COMZ	6 (23.1%)	10 (38.5%)	0.367
	Le Fort I and II	5 (19.2%)	9 (34.6%)	0.348
Fracture types	Le Fort III	3 (11.5%)	0 (0%)	0.465
	Anterior Skull base	2 (7.7%)	4 (15.4%)	0.664
	NOE complex	8 (30.8%)	3 (11.5%)	0.174
	Nasal Bone	6 (23.1%)	0 (0%)	0.03
	Mandible	3 (11.5%)	3 (11.5%)	0.862

**Table 2:** Comparison of procedure duration, intraoperative and postoperative complications, infection rates, and mean hospitalization time between submental intubation and tracheostomy.

		Submental Intubation	Tracheostomy	*p*-Value
Procedure mean time (minutes)		9.76 ± 2.3	17 ± 4.2	<0.001
	Total	2	5	0.049
Intraoperative complications	Balloon damage	1 (3.8%)	0 (0%)	
	Subcutaneous emphysema	0 (0%)	2 (7.7%)	
	Thyroid gland injury	0 (0%)	1 (3.8%)	
	Hematoma	1 (3.8%)	0 (0%)	
	Hemorrhage	0 (0%)	2 (7.7%)	
	Total	3	6	0.037
Postoperative complications	Hypertrophic scar	1 (3.8%)	0 (0%)	
	Submental swelling	2 (7.7%)	1 (3.8%)	
	Tracheomalacia	0 (0%)	3 (11,5%)	
	Scar dehiscence	0 (0%)	2 (7.7%)	
Infection rates		1 (3.8%)	8 (30.8%)	0.028
Mean hospitalization duration (days)		11.5 ± 3.29	23.96 ± 6.47	<0.001

## Data Availability

The data generated and analyzed during this study are not publicly available due to institutional and privacy policies but are available from the corresponding author upon reasonable request.

## Abbreviations

The following abbreviations are used in this manuscript:

IMF	Intermaxillary fixation
NOE	Naso-orbital-ethmoid
COMZ	Zygomaticomaxillary complex
SMI	Submental intubation
